# Raising Our Voices: The Impact of a Dementia Choir on Well-Being and Quality of Life

**DOI:** 10.1093/geroni/igaa057.3034

**Published:** 2020-12-16

**Authors:** Debra Sheets, Stuart MacDonald, Andre Smith

**Affiliations:** University of Victoria, Victoria, British Columbia, Canada

## Abstract

Stigma represents one of the biggest barriers to living well with dementia following diagnosis. Social isolation is common as roles, friendships and opportunities to participate in the broader community disappear. An intergenerational dementia choir is a joyful activity that offers opportunities for learning, friendships and purposeful engagement towards common goals (e.g., regular social engagement, public concerts at season’s end). Data collection involved surveys and interviews with 32 dyads comprised of persons with dementia (PwD) and caregivers, as well as focus groups with 29 high school students. Results illustrate the development of a choir community across weeks of participation with far reaching benefits. Both caregivers and PwD experienced reductions in health risks and improvements in quality of life. Students’ understanding of dementia became more positive over time and new friendships developed. The discussion focuses on the need for meaningful and inclusive community activities for PwD and their caregivers.

